# Metastasizing tenosynovial giant cell tumour, diffuse type/pigmented villonodular synovitis

**DOI:** 10.1186/s13569-015-0030-2

**Published:** 2015-06-06

**Authors:** A Righi, M Gambarotti, M Sbaraglia, T Frisoni, D Donati, D Vanel, A P Dei Tos

**Affiliations:** Department of Pathology, Istituto Ortopedico Rizzoli, Via del Barbiano 1/10, 40136 Bologna, Italy; Oncologic Department, Rizzoli Orthopaedic Institute, Bologna, Italy; Department of Pathology, Treviso Regional Hospital, Treviso, Italy

**Keywords:** Tenosynovial giant cell tumor, Diffuse type, Lymph node, Metastases

## Abstract

Tenosynovial giant cell tumour, diffuse type, also known under a variety of other terms including diffuse pigmented villonodular synovitis, tends to be locally aggressive and not infrequently can show multiple recurrences. The differential diagnosis with the extremely rare and somewhat controversial malignant variant of tenosynovial giant cell tumour, diffuse type, is challenging due to overlapping radiologic features of these two entities. Malignant tenosynovial giant cell tumour is defined by the presence of overtly malignant sarcomatous areas. We describe a very unusual case of a 63-year-old man affected by tenosynovial giant cell tumour, diffuse type of the knee that, despite absence of morphologic evidence of sarcomatous transformation, developed inguinal lymph node metastases following multiple surgical procedures.

## Background

Tenosynovial giant cell tumour, diffuse type (D-TGCT), also alternatively labeled as diffuse type giant cell-tumour, or diffuse pigmented villonodular synovitis, usually occurs in tendon sheaths and in the synovia of the large joint. The 2013 WHO Classification of Tumors of Soft Tissue and Bone classifies D-TGCT as a locally aggressive neoplasm characterized by high rate of local recurrences [1].

The pathogenetic mechanisms underlying D-TGCT have been source of sharp debate. Some authors have considered it as an inflammatory disorder [2, 3], however, based on demonstration of monoclonality, currently there exists broad consent in considering D-TGCT as a true neoplasm, [1–9]. Cytogenetic studies have demonstrated that translocations of chromosome 1, involving *CSF1* gene, represent frequent alterations that set the rationale for effective treatment with imatinib [8].

The development of metastatic spread, to the lungs, of histologically benign D-TGCT (overlapping morphologically with the primary tumor) represents an exceptionally rare reported event. This occurrence should be differentiated from true malignant variants of this entity [1, 5, 7, 9]. We report a D-TGCT occurring in the knee joint of a 63-year-old man who, despite absence of malignant sarcomatous evolution, developed multiple lymph node metastases after multiple surgeries, the first of which had occurred 28 years before.

## Case report

In March 2012, a caucasian 63-year-old man was seen in our institution for a painful swelling of the right knee joint present since 2 years. Pain was exacerbated by digital pressure. This patient resulted affected by D-TGCT that had been surgically treated with multiple synovectomies in 1987, 1990 and 1996, respectively. Histologic review of the original specimens of the primary tumor and of all recurrences was performed. All the specimens exhibited overlapping morphology by showing a combination of hyperplastic synovia, a mononuclear cell proliferation organized in a nodular pattern of growth, scattered hemosiderin-laden macrophages, xanthoma cells and multinucleated giant cells. These findings met the diagnostic criteria for D-TGCT and we therefore confirmed the primary diagnosis. Because of pain and persistent joint effusion, the patient underwent another synovectomy at our institution, and the histologic evaluation of the specimen confirmed the diagnosis of D-TGCT without atypical features. In contrast with previous samples a greater amount of fibrosis was seen.

**Figure 1:**
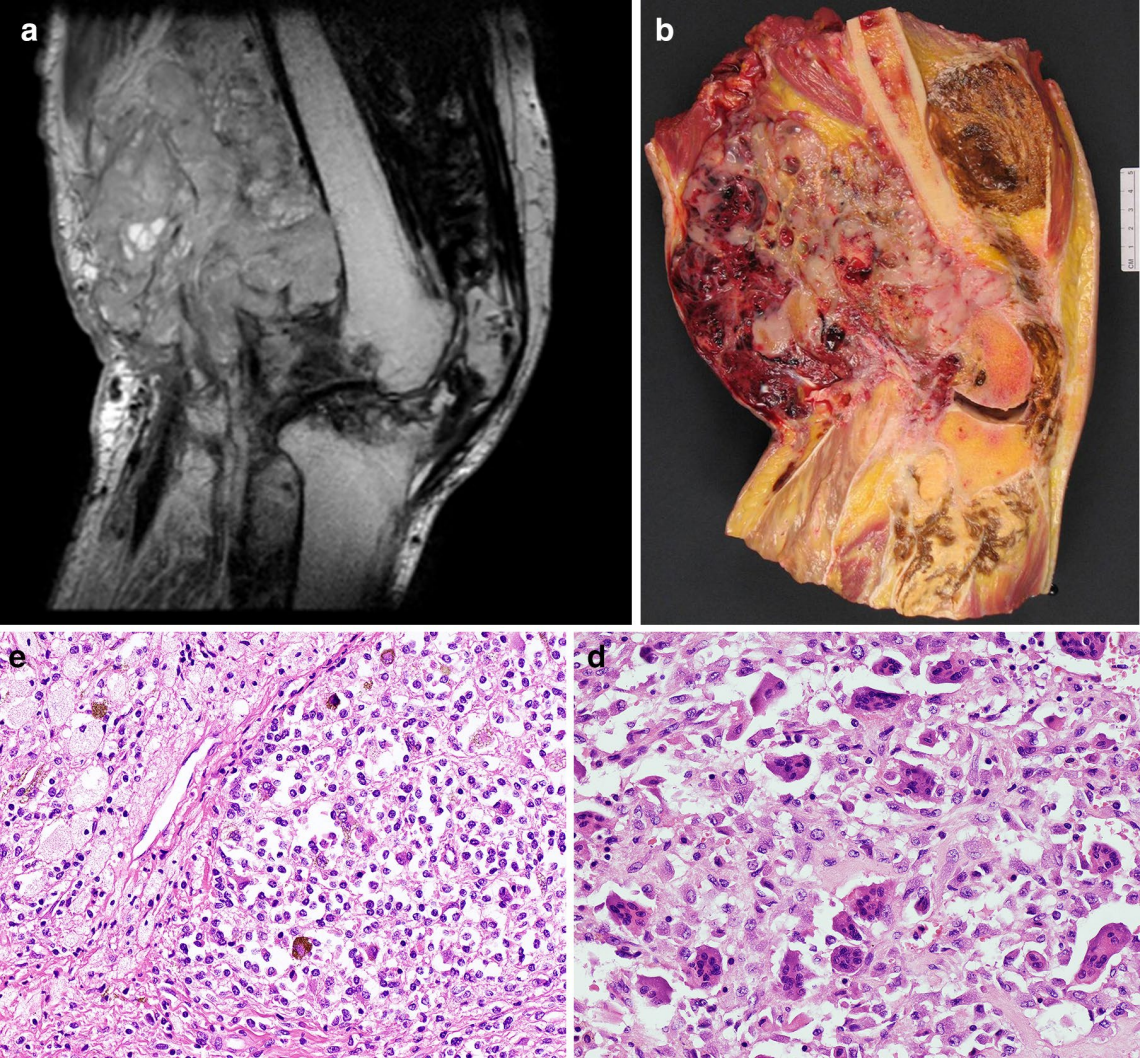
**a** On magnetic resonance imaging, an heterogeneous multinodular mass was observed on sagittal T1-weighted images (TR/TE: 1,200/120). Dark signal nodules, compatible with hemosiderin deposition, were identified. **b** On macroscopy a multinodular lesion was evident in the leg and in the thigh showing variegated colour. **c**, **d** On haematoxylin and eosin staining the lesion showed synovial-like mononuclear cells without cytologic atypia, admixed with multinucleate giant cells.

**Figure 2:**
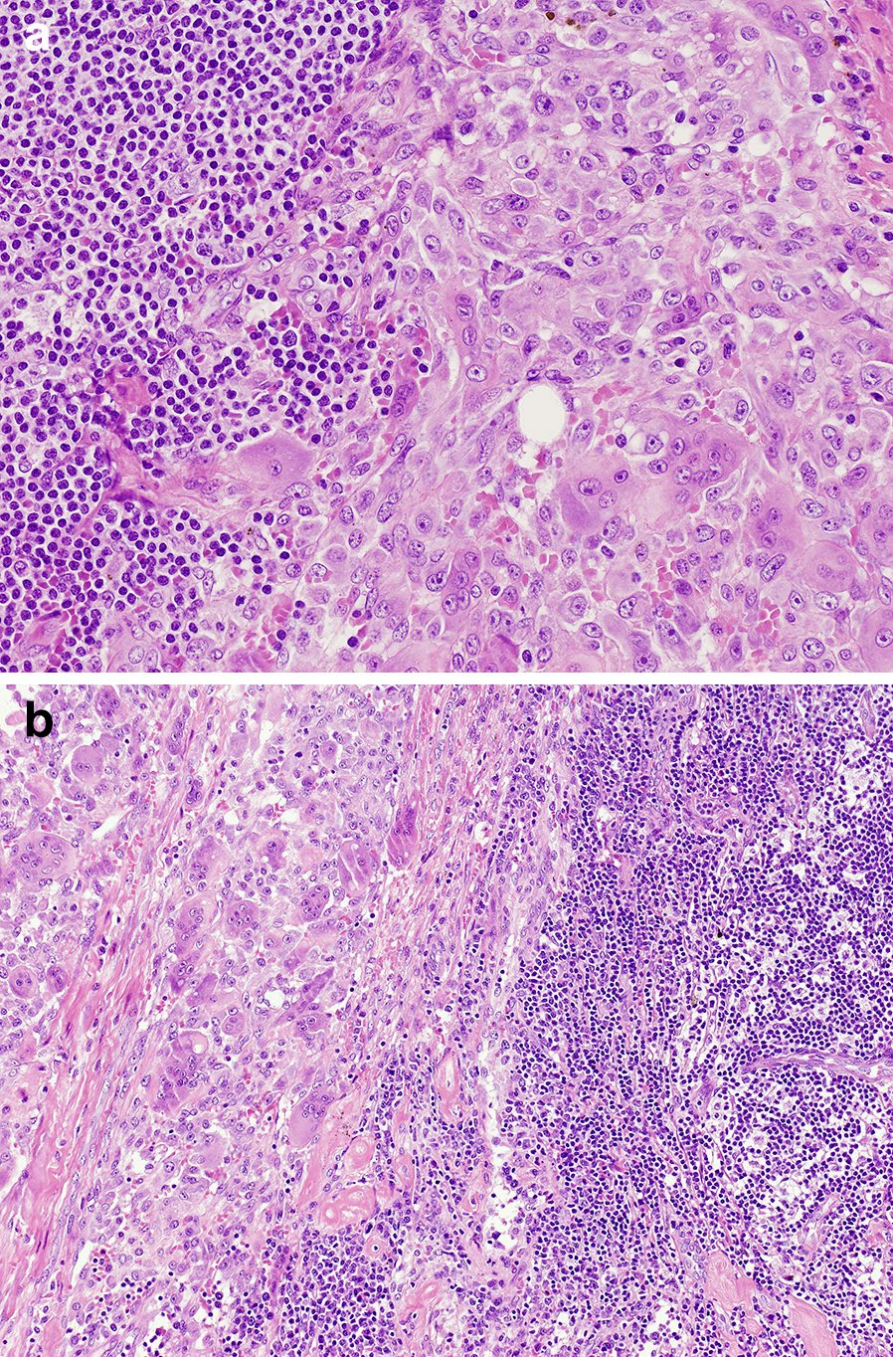
**a**, **b** The haematoxylin and eosin staining of the inguinal lymph node revealed a metastasis of D-TGCT showing a proliferation of synovial-like mononuclear cells, admixed with multinucleate giant cells and siderophages.

Two years later the patient presented again with painful swelling of the right knee associated with pain localized in the right hip. Loss of weight and functional limitation of the right leg was observed. Magnetic nuclear resonance imaging demonstrated extra-articular soft tissue multilobular masses around the posterior part of the knee joint, with erosion of the lateral femoral condyle. Extension into the patello-femoral joint and the proximal tibia with a low signal intensity on T2-weighted image was present. In T1-weighted images, dark signal nodules, compatible with hemosiderin deposition, were readily identified (Figure 1a). Clinical examination showed recurrent tumour in the knee whereas computed tomography imaging revealed pelvic lymph nodes enlargement. In consideration of the extensive destruction with consequent functional compromise of the knee joint the patient underwent an “above the knee” amputation. During surgical procedure frozen examination of one inguinal lymph node was performed that showed a sub-capsular proliferation of mononucleated cells, associated with multinucleated giant cells and siderophages consistent with lymph node seeding of D-TGCT (Figure 2a, b).

Macroscopically, the evaluation of the knee disarticulation revealed a multinodular lesion of 30 cm × 25 cm × 21 cm, that was extensively infiltrating both the leg and the thigh with massive destruction of the knee joint. Alternation of white, yellowish and brownish areas with hematic-cystic lesions was seen (Figure 1b). Histological analysis of the lesion showed the same morphological features as the previous samples and confirmed the diagnosis of D-TGCT. In particular, the lesion showed synovial-like mononuclear cells lacking significant cytologic atypia, admixed with multinucleated giant cells (Figure 1c, d). Foci of ischemic necrosis were also evident. The mitotic rate was lower than two figures per 10 high power field. Ki67 proliferative index did not exceed 5% of neoplastic cells.

No areas of malignant transformation were identified in the specimen. Four months later a CT scan of the pelvis revealed a mass located in the soft tissue of the contralateral buttock, associated with inguinal lymph nodes swelling without evidence of lung metastases. In January 2015, the patient started systemic treatment with imatinib.

## Discussion

Metastatic spread to the lungs and/or to locoregional lymph nodes represents a very unusual event in D-TCGT [7]. Such occurrence in principle should prompt reconsideration of the original diagnosis [1, 5]. The clinical differential diagnosis between D-TCGT and its malignant variant is very difficult, due to overlapping radiologic features [7, 10]. The designation malignant D-TGCT is used for lesions in which a typical-appearing benign D-TGCT coexists with overtly malignant areas, or when the typical benign D-TGCT recurs as a morphologically malignant mesenchymal lesions [7]. The criteria of malignancy for D-TCGT were defined by Bertoni et al. [5] who reported the presence of larger mononuclear cells, featuring round or round to oval hyperchromatic nuclei sometimes showing prominent nucleoli, and presence of atypical mitotic figures. Xanthomatous cells as well as giant cells decreased in number and sometimes absent. A nodular pattern of growth was most often seen. In some cases a gradient of differentiation (zoning phenomenon) between the periphery and the central part of the nodules was observed [5]. To our knowledge, 32 cases of malignant D-TCGT are reported in literature: 17 cases were primary malignant D-TGCT and 15 arose from a prior histologically benign lesion [1–6, 9]. Seven out of these 32 cases metastasized to regional lymph node: four patients died of disease with a rapid progression (mean 22 months, range 12–41 months), two were alive with disease 17, and 36 months after the first surgical treatment, respectively and only one patient was well without disease 10 months after the first surgical treatment. None of the above described morphologic features were observed in our case. However, despite absence of histologic features of malignancy lymph node metastases developed 28 years since first surgical procedure. The only two other cases reported in the literature describe a 50-year-old man with D-TGCT in the popliteal fossa of the knee that gave rise, after two local recurrences, to inguinal lymph node metastases 56 months after the first surgical treatment [9], and a 44-year-old male with D-TGCT in the knee joint that developed, after multiple local recurrences, lymph nodes and lung metastases 9 years after the first surgical treatment [4]. The morphologic features of our case are similar to those previously reported by Somerhausen and Fletcher [9] and Asano et al. [4].

The mechanism responsible for distant metastases in D-TGCT has not been established yet [1, 4, 7]. It has been speculated that lymph node metastases might result from passive rather than active vascular involvement by the tumor, possibly prompted by repeated surgical manipulation [4]. A similar process has been postulated to explain lung spread in other locally aggressive neoplasm such as giant cell tumor of the bone and chondroblastoma [11].

Regional lymph node metastases of other benign soft tissue neoplasms have also been reported [12]. Uterine leiomyoma is an example in which the term benign metastasizing smooth muscle tumor is used to describe cytologically bland, mitotically inactive smooth muscle neoplasms occurring in the lymph nodes or lungs of patients with benign-looking uterine smooth muscle tumors [12].

We herein report a case of conventional D-GCT of the knee that, despite lacking any morphologic features of malignancy developed bilateral loco-regional lymph-node metastasis.

Even if this represents an extremely rare occurrence, long term follow-up is strongly advised, particularly for those patients experiencing multiple local recurrences.
